# Integrative analysis of chromatin accessibility and transcriptome landscapes in the induction of peritoneal fibrosis by high glucose

**DOI:** 10.1186/s12967-024-05037-6

**Published:** 2024-03-05

**Authors:** Qiong Song, Pengbo Wang, Huan Wang, Meijing Pan, Xiujuan Li, Zhuan’e Yao, Wei Wang, Guangbo Tang, Sen Zhou

**Affiliations:** 1 Department of Nephrology, Shaanxi Second People’s Hospital, Xi’an, Shaanxi People’s Republic of China; 2 Department of Nephrology, The First Hospital of Lanzhou University, Lanzhou, Gansu People’s Republic of China; 3 Key Laboratory of Biomedical Information Engineering of Ministry of Education, School of Life Science and Technology, Xi’an Jiaotong University, Xi’an, Shaanxi People’s Republic of China; 4 Department of Emergency, Xijing Hospital, The Fourth Military Medical University of People’s Liberation Army, Xi’an, Shaanxi People’s Republic of China; 5 School of Automation Science and Engineering, Faculty of Electronic and Information Engineering, Xi’an Jiaotong University, Xi’an, Shaanxi People’s Republic of China; 6 Department of Clinical Medicine, Xi’an Medical University, Xi’an, Shaanxi People’s Republic of China

**Keywords:** End-stage kidney disease, High glucose, Peritoneal fibrosis, Chromatin accessibility, Transcriptome landscapes, HIF-1α, TGF-β1

## Abstract

**Background:**

Peritoneal fibrosis is the prevailing complication induced by prolonged exposure to high glucose in patients undergoing peritoneal dialysis.

**Methods:**

To elucidate the molecular mechanisms underlying this process, we conducted an integrated analysis of the transcriptome and chromatin accessibility profiles of human peritoneal mesothelial cells (HMrSV5) during high-glucose treatment.

**Results:**

Our study identified 2775 differentially expressed genes (DEGs) related to high glucose-triggered pathological changes, including 1164 upregulated and 1611 downregulated genes. Genome-wide DEGs and network analysis revealed enrichment in the epithelial–mesenchymal transition (EMT), inflammatory response, hypoxia, and TGF-beta pathways. The enriched genes included *VEGFA*, *HIF-1α*, *TGF*-*β1*, *EGF*, *TWIST2*, and *SNAI2*. Using ATAC-seq, we identified 942 hyper (higher ATAC-seq signal in high glucose-treated HMrSV5 cells than in control cells) and 714 hypo (lower ATAC-seq signal in high glucose-treated HMrSV5 cells versus control cells) peaks with differential accessibility in high glucose-treated HMrSV5 cells versus controls. These differentially accessible regions were positively correlated (R = 0.934) with the nearest DEGs. These genes were associated with 566 up- and 398 downregulated genes, including *SNAI2*, *TGF*-*β1*, *HIF-1α*, *FGF2*, *VEGFA*, and *VEGFC*, which are involved in critical pathways identified by transcriptome analysis. Integrated ATAC-seq and RNA-seq analysis also revealed key transcription factors (TFs), such as *HIF-1α*, *ARNTL*, *ELF1*, *SMAD3* and *XBP1*. Importantly, we demonstrated that HIF-1α is involved in the regulation of several key genes associated with EMT and the TGF-beta pathway. Notably, we predicted and experimentally validated that HIF-1α can exacerbate the expression of TGF-β1 in a high glucose-dependent manner, revealing a novel role of HIF-1α in high glucose-induced pathological changes in human peritoneal mesothelial cells (HPMCs).

**Conclusions:**

In summary, our study provides a comprehensive view of the role of transcriptome deregulation and chromosome accessibility alterations in high glucose-induced pathological fibrotic changes in HPMCs. This analysis identified hub genes, signaling pathways, and key transcription factors involved in peritoneal fibrosis and highlighted the novel glucose-dependent regulation of TGF-β1 by HIF-1α. This integrated approach has offered a deeper understanding of the pathogenesis of peritoneal fibrosis and has indicated potential therapeutic targets for intervention.

**Supplementary Information:**

The online version contains supplementary material available at 10.1186/s12967-024-05037-6.

## Introduction

Peritoneal dialysis (PD) is an appealing treatment option for patients with end-stage kidney disease (ESKD) [1]. Presently, PD constitutes approximately 10% of the global modalities employed for renal replacement therapy [2]. However, long-term peritoneal dialysis can lead to functional and structural changes in the peritoneal membrane, resulting in peritoneal fibrosis. Currently, the specific mechanism underlying peritoneal fibrosis has not been determined. Consequently, peritoneal fibrosis represents a significant challenge for long-term peritoneal dialysis patients [3].

Peritoneal fibrosis (PF) is believed to arise from various factors, including the use of bioincompatible dialysates (high glucose, acidic solutions, and glucose degradation products), uremia, peritonitis, and chronic inflammation [4]. Among these factors, high glucose is considered a major trigger for peritoneal fibrosis. Glucose is the primary osmotic agent utilized due to its high effectiveness, cost-effectiveness, and satisfactory safety profile. However, to establish an osmotic gradient for the removal of electrolytes and toxins through convection with water, glucose is employed at concentrations 10 to 50 times greater than that of serum. This represents the primary challenge associated with the bioincompatibility of peritoneal dialysis solutions. Elucidating the fundamental mechanisms that connect high glucose-induced peritoneal fibrosis would constitute a significant breakthrough in the efficient management of ESKD.

Moreover, peritoneal mesothelial cells play a central role in peritoneal fibrosis associated with peritoneal dialysis [5]. Although numerous studies have illuminated the intricate mechanisms involved in high glucose-induced peritoneal fibrosis, including the critical role of TGF-β1 as a mediator and epithelial–mesenchymal transition (EMT) as a key process in initiating peritoneal fibrosis, a comprehensive and systematic characterization of gene expression changes in human peritoneal mesothelial cells (HPMCs) under high glucose conditions is lacking. Understanding the cellular and molecular mechanisms that contribute to peritoneal membrane fibrosis could be valuable in developing therapies designed to mitigate deterioration and restore homeostasis in the peritoneal membrane.

Epigenetic alterations play a crucial role in the regulation of gene expression, while open accessible regions on chromosomes serve as binding sites for transcription factors and other regulatory elements. These dynamic changes in chromosome accessibility occur in various biological processes, including cell differentiation, development, response to external stimuli, and pathological changes. ATAC-seq is a newly developed method that allows the evaluation of chromosome accessibility. The process relies on effective enzymatic cleavage and transposition of Tn5 to identify open chromatin regions [6]. This technique has been widely used in diverse disease-associated studies to elucidate the landscape of chromatin accessibility, alterations, and transcriptional regulators that play vital roles in disease pathogenesis [7–10]. However, the dynamics of chromosome accessibility and its role in the regulation of gene expression in high glucose-triggered PF have not been extensively investigated.

In this study, we utilized ATAC-seq in combination with RNA-seq to characterize the landscape and examine the relationship between chromatin accessibility and gene transcription in high glucose-treated HMrSV5. By analyzing DEGs, we systematically identified key genes and core molecule interaction networks involved in high glucose-triggered PF. Moreover, the analysis of differentially accessible regions (DARs) enabled us to identify regulatory DNA sequences and core TFs that may be responsible for the observed changes during this process.

## Methods

### Cell culture

HMrSV5 and HEK293T cells were cultured in high-glucose DMEM (Gibco/Thermo Fisher Scientific) supplemented with 10% FBS (HyClone) and 1/100 (vol/vol) penicillin/streptomycin (Gibco/Thermo Fisher Scientific) at 37 °C in a humidified incubator with 5% CO2. For the extra high glucose treatment, cells were cultured in high glucose DMEM supplemented with an additional 60 mM glucose.

### Western blot

Total protein was extracted from each group via RIPA lysis buffer (Beyotime, P0013B) containing 2 mM PMSF (Beyotime, ST506). The resulting cell lysates were subjected to centrifugation at 12,000 × g and 4 °C for 10 min. The total protein was then combined with 5 × SDS‒PAGE loading buffer and boiled at 100 °C for 10 min. Following subsequent centrifugation at 12,000 rcf for 10 min, the supernatants were collected for SDS‒PAGE electrophoresis. Equal amounts of protein (40 μg/lane) from each sample were loaded onto a 10% SDS‒PAGE gel and transferred to a 0.45 μm polyvinylidene difluoride membrane. The gel was blocked with 5% nonfat dry milk in Tris-buffered saline containing 0.1% Tween-20 (TBST) at room temperature for 2 h. Subsequently, primary antibodies (Vimentin, ABclonal, A19607, 1:1000; E-cadherin, ABclonal, A20798, 1:1000; β-actin, Cell Signaling, 4970, 1:2000) were incubated overnight at 4 °C. After washing three times with 1 × TBST solution (5 min each), the membranes were incubated with an HRP-conjugated goat anti-rabbit secondary antibody (ABclonal, AS041, 1:5000) for 1 h at room temperature. After an additional three washes with 1 × TBST, the bands were visualized using BeyoECL reagents (Beyotime, P0018S).

### Immunofluorescence staining

HMrSV5 cells were plated in a 24-well plate at a density of 8 × 10^4^ cells per well. After incubation with either normal culture medium or culture medium supplemented with 60 mM glucose, the cells were washed with PBS three times. Subsequently, the cells were fixed with ice-cold methanol for 10 min, followed by another three washes with PBS. The cells were then incubated in PBS containing 0.5% Triton X-100 at room temperature for 30 min and blocked with 1% BSA at room temperature for 1 h. Next, the cells were incubated with primary antibodies against Vimentin (ABclonal, A19607) overnight at 4 °C (1:200). The cells were then rinsed three times with PBS and incubated with secondary antibodies in the dark at room temperature for 30 min [goat anti-rabbit IgG (Alexa Fluor^®^ 488) (1:500)]. After an additional three washes with PBS, the cells were mounted with mounting medium containing Hoechst 33,342. Subsequently, the cellular morphology was observed, and images were captured under a fluorescence microscope.

### Construction of the *HIF-1α* overexpression plasmid

RNA was extracted from HMrSV5 cells using TRIzol (Thermo Fisher Scientific, 15596026), and the resulting RNA was subsequently used as an input to produce cDNA via the PrimeScript 1st Strand cDNA Synthesis Kit (Takara, 6110A). The coding sequence of *HIF-1α* was amplified with Q5 DNA polymerase (NEB, M0491S) using cDNA as a template. Subsequently, the amplified *HIF-1α* fragment was subcloned and inserted into the PLVX-IRES-puro vector using the ClonExpress Ultra One Step Cloning Kit (Vazyme, C115) to generate the expression plasmid. The primers used for *HIF-1α* amplification were as follows: sense: 5ʹ-ggatctatttccggtgaattcATTCACCATGGAGGGCGC-3ʹ, antisense: 5ʹ-ggagggagaggggcgggatccTCAGTTAACTTGATCCAAAG-CTCTG-3ʹ.

### Lentivirus production and transduction

The *HIF-1α*-overexpressing and empty viruses were generated by transfecting the constructs with pMD2.G and psPAX2 into HEK293T cells using Lipo6000 (Beyotime, C0526). After 12 h of transfection, the medium was replaced with fresh medium. The virus was harvested 48 h later, and the supernatant was collected by centrifugation at 4000 rcf for 10 min and filtered through a 0.45 μm filter unit (Millipore). The cells were transduced with the recombinant lentivirus for 24 h and then selected with puromycin for 1 week, starting 48 h after transduction.

### Luciferase activity assay

A 2000-bp promoter fragment surrounding the *TGF-β1* transcription start site was amplified from genomic DNA and subsequently cloned and inserted into the pGL3-Basic vector. The sequences of primers used were as follows: Sense: 5ʹ-atctgcgatctaagtaagcttCGCAGGGTGTTGAGTGACAGGAG-3ʹ, Anti-sense: 5ʹ-cagtaccggaatgccaagcttGGTGACCTCCTTGGCGTAGTAGTCG-3ʹ. Following construction, these plasmids were transfected into HMrSV5 cells using TransIntro PL Transfection Reagent (TransGen). Concurrently, the phRL plasmid containing Renilla luciferase (Promega) was introduced as an internal control. The culture medium was changed to either normal or 60 mM glucose-containing medium 24 h posttransfection. After a 3 day incubation, luciferase activity, with the Renilla luciferase vector phRL serving as the internal reference for luciferase signals, was assessed using the Dual-Luciferase Reporter Assay System (Beyotime, RG042M).

### ELISA

The HMrSV5-OE control and HMrSV5-OE *HIF-1α* cells were plated in a 24-well plate at a density of 8 × 10^4^ cells/well, cultured overnight, and subsequently exposed to either normal culture medium or culture medium supplemented with 60 mM glucose. After a 3 day incubation, the culture medium was collected, followed by centrifugation for 10 min at 1000 rcf. The resulting supernatant was utilized for TGF-β1 concentration assessment using the Transforming Growth Factor Beta 1 ELISA Kit (ABclonal, RK00055) following the kit protocol. In brief, the samples were activated by adding 10 μL of 1 N HCl to 40 μL of supernatant, mixing for 10 min at room temperature, and then neutralizing with 10 μL of 1.2 N NaOH/0.5 M HEPES. The activated samples were diluted 20-fold. Subsequently, 100 μL of standard TGF-β1 or sample dilutions was added to ELISA strips precoated with anti-TGF-β1 antibody and incubated at 37 °C for 2 h. The strips were washed three times with wash buffer. Next, 100 μL of biotin-conjugated antibody was added, followed by a 1 h incubation at 37 °C. After another three washes with wash buffer, 100 μL of working streptavidin-HRP was added, and the mixture was incubated at 37 °C for 30 min. Following three washes with wash buffer, 100 μL of TMB substrate was added, and the mixture was incubated for 20 min at 37 °C. Finally, 50 μL of stop solution was added, and the optical density of each well was measured using a microplate reader at 450 nm. The TGF-β1 concentration was calculated based on the standard sample curve.

### RNA-seq

Total RNA from HMrSV5 cells was extracted using TRIzol Reagent (Thermo, 15596026). Subsequently, the extracted RNA was further purified using an RNeasy Mini Kit (Qiagen, 74,004). RNA-Seq was performed on the purified RNA following a previously described method [10].

### ATAC-seq

ATAC-seq was performed as previously described, with slight modifications [11]. Briefly, HMrSV5 cells were digested with 0.25% trypsin, and after collection and washing with ice-cold PBS, 1 × 10^5^ cells were lysed in ice-cold lysis buffer (10 mM NaCl, 3 mM MgCl2, 0.5% IGEPAL CA-630, 0.1% Tween-20, and 10 mM Tris–HCL, pH 7.5) for 15 min on ice. The lysate was then centrifuged at 800 rcf for 5 min at 4 °C. After discarding the supernatant, the nuclei were collected, and reaction buffer containing Tn5 transposase (Vazyme Biotech, TD501) was added. The reactions were performed at 1200 rpm and 37 °C in a thermomixer for 30 min. The transposed DNA fragments were purified using the Qiagen MinElute PCR Purification Kit (QIAGEN, 28004). Subsequently, the purified DNA fragments were amplified using KAPA HotStart ReadyMix (Kapa Biosystems, KM2605) following the manufacturer’s instructions. Next, Agencourt AMPure XP (Beckman, A63881) was used to purify the amplified DNA library. Finally, the library DNA was sequenced with 150 paired-end reads using an Illumina HiSeq X10 instrument.

### RNA-Seq data analysis

The sequence files of the RNA-seq data were aligned to the human genome version hg38 using HISAT2 [12] (version 2.2.1). Subsequently, stringtie [13] (version 2.1.5) was used to call read counts and calculate TPM (transcripts per million) values. For the differential gene expression analysis, DESeq2 [14] in the R environment was used, and the read counts were used as input. The cutoff values for identifying differentially expressed genes were set at a fold change ≥ 2 and p value < 0.05. Genes meeting these criteria were considered to be differentially expressed between the experimental groups.

### Data analysis and visualization of ATAC-seq data

For ATAC-seq data, reads were trimmed by Trimmomatic [15]and subsequently aligned to the human GRCh38 genome using Bowtie2 [16] (version 2.4.2). Duplicated reads were removed by Picard MarkDuplicates (version 2.25.0), and only uniquely mapped reads were retained for further analysis. The mitochondrial DNA and low-quality mapped reads (MAPQ score < 10) were filtered out using SAMtools [17] (version 1.12). MACS2 [18] (version 2.2.7.1) with the following parameters were used to call ATAC-seq peaks: –shift 100 –extsize 200 –nomodel –B –SPMR –g hs. The reproducible peaks of two replicates were produced by IDR (version 2.0.4.2). The read coverages of the genomic regions in the BAM files were determined using Bedtools multicov [19] (version 2.30.0). DiffBind [20] was used for differential analysis of the ATAC-seq peaks. The annotation of the peaks in the region proximal to the target genes was performed via the ChIPseeker package (version 1.28.3) in the R environment [21]. GREAT (http://great.stanford.edu/) was used to annotate the peaks in the long-distance region. To visualize continuous ATAC-seq signals, bigwig files were created with deeptools bamCoverage [22] (version 3.5.1). The visualization of bigwig files was achieved using IGV [23].

### Prediction of key TFs

ANANSE [24] (version 0.3.0) was used to predict key TFs. Briefly, BAM files from ATAC-seq, RNA expression files and different RNA expression files were used as inputs. The compartments of ANANSE, ananse binding, ananse network, and ananse influence, were determined in sequence.

### GO and KEGG enrichment analysis

GO and KEGG analyses were performed by Metascape [25] (https://metascape.org/gp/index.html#/main/step1).

### Connectivity map (CMap) analysis

CMap (https://clue.io) serves as a gene expression profile database, employing gene expression signature interventions to unveil connections between diseases, genes, and small molecule compounds. In this study, upregulated and downregulated hub genes were subjected to analysis via the CMap online database to identify potential small-molecule drugs for PF treatment. Compounds displaying negative connectivity scores were then selected for subsequent analysis, including molecular docking.

### Molecular docking

Protein 3D structures were retrieved from either https://alphafold.ebi.ac.uk/ or RCSB (https://www.rcsb.org/), while the 3D structures of drugs were obtained from the PubChem database [26]. The drug structures were subsequently converted into Mol2 format using Open Babel software [27]. In cases where water was present in the protein 3D structure file, it was removed using PyMOL. Hydrogen atoms and charges were added using Autodock GUI. Subsequently, molecular docking of the drugs and target proteins was conducted using Autodock GUI, and the results were visualized with PyMOL.

### Statistical analysis

All analyses were performed using GraphPad Prism (version 9.0) or R (https://www.r-project.org/). The data are presented as the mean ± s.d. Two-tailed unpaired or paired Student’s t test, ANOVA (one-way or two-way), and Spearman correlation coefficient were calculated according to the type of experiment. *P* < 0.05 was considered to indicate statistical significance.

## Results

### Transcriptome alterations associated with high glucose treatment in HMrSV5 cells.

**Fig. 1:**
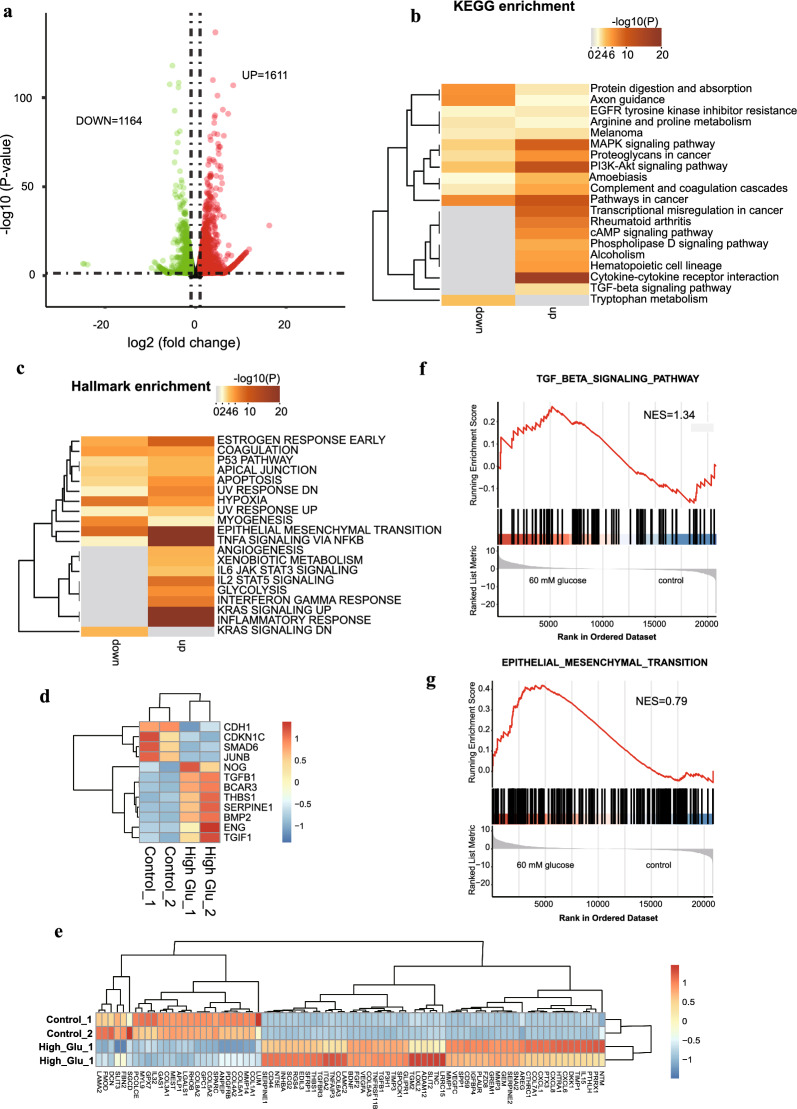
Transcriptome alterations in high glucose–treated HMrSV5 cells. **a** Volcano plots illustrating the DEGs detected by RNA-seq. The data were filtered by a *P* value < 0.05 and an absolute fold change > 2. **b**, **c** KEGG enrichment (**b**) or hallmark gene set enrichment (**c**) of upregulated DEGs and downregulated DEGs. Up/Down: upregulated or downregulated genes in high glucose-treated HMrSV5 cells. **d**, **e** Heatmap showing dysregulated genes associated with hallmark of epithelial mesenchymal transition (**d**) or the TGF-beta signaling pathway. **f**, **g** Gene Set Enrichment Analysis (GSEA) shows that the TGF-beta signaling pathway (**f)** and Epithelial mesenchymal transition hallmark gene set (**g**)  were enriched in high-glucose treated HMrSV5 cells

To explore potential genes associated with high glucose-containing peritoneal dialysate treatment, we conducted RNA-seq analysis of HMrSV5 cells cultured in standard culture medium or culture medium supplemented with 60 mmol/L glucose. A total of 2775 DEGs were differentially expressed in response to high-glucose treatment, with 1164 genes downregulated and 1611 genes upregulated compared to those in the control condition (Fig. 1a, Additional file 5: Supplementary table 1). To gain insight into the crucial pathways involved in the glucose response in HMrSV5 cells, KEGG enrichment analysis was employed. The upregulated genes were mainly associated with the MAPK signaling pathway, the PI3K-Akt signaling pathway, cytokine‒cytokine receptor interactions, and the TGF-beta pathway (Fig. 1b). However, the downregulated DEGs were significantly enriched in pathways related to protein digestion and absorption, as well as axon guidance (Fig. 1b). Additionally, enrichment analysis of the hallmark gene sets revealed that the upregulated DEGs were predominantly enriched in processes such as EMT, TNF-α signaling via NFKB, angiogenesis, glycolysis, KRAS signaling, and the inflammatory response. Moreover, the downregulated DEGs were associated with processes such as myogenesis and KRAS signaling (Fig. 1c). Moreover, gene ontology analysis of biological processes indicated that the upregulated DEGs were enriched in chemotaxis, regulation of adhesion, regulation of epithelial cell proliferation, and inflammatory response (Fig. S1a). On the other hand, the downregulated DEGs were specifically involved in processes related to external encapsulating structure organization (Additional file 1: Fig. S1a).

EMT plays a central role in the development of fibrosis induced by high glucose treatment in HMrSV5 cells. To further investigate this phenomenon, we assessed the expression of classic EMT-associated TFs, which include *TWIST1*, *TWIST2*, *ZEB1*, *ZEB2*, *SNAI1*, and *SNAI2* [28–31]. Interestingly, we observed significant overexpression of *TWIST2* and *SNAI2*, suggesting their potential central role in high glucose-induced EMT and fibrosis in HMrSV5 cells (Additional file 1: Fig. S1b). Moreover, 56 genes and 27 genes in the hallmark epithelial–mesenchymal transition gene sets were upregulated and downregulated, respectively. The genes included *CXCL1*, *CXCL6*, *CXCL8*, *SNAI2*, *SPP1*, *VEGFA*, *VEGFB*, *SERPINE1*, *TGF*-*β1*, and *THBS1* (Fig. 1e). It has been reported that cytokines can promote EMT [32], and in our study, we also noticed that upregulated genes were highly enriched in cytokine‒cytokine receptor interactions. These findings led us to investigate the expression of several EMT-promoting extracellular factors. Our findings revealed that, in addition to *TGF*-*β1*, *TGF*-*β2*, *FGF2*, *FGF1*, *FGF5*, and *FGF7* were also upregulated, indicating the complexity of the EMT-promoting mechanism (Additional file 1: Fig. S1c). Furthermore, given the significant role of TGF-β1 in high glucose-triggered EMT in HPMC cells, we conducted a detailed analysis of dysregulated genes in the TGF-beta pathway. The results revealed that *TGF*-*β1*, *NOG*, *BCAR3*, *THBS1*, *SERPINE1*, *BMP2*, *ENG*, and *TGIF1* were upregulated, while *CDH1*, *CDKN1C*, *SMAD6*, and *JUNB* were downregulated (Fig. 1d). Finally, GSEA revealed activation of the TGF-beta signaling pathway and the hallmark of epithelial–mesenchymal transition (EMT) in HMrSV5 cells treated with high glucose (Fig. 1f, g).

Overall, our findings shed light on the intricate interplay among various factors, transcription factors, and pathways involved in high glucose-induced EMT and fibrosis in HMrSV5 cells, helping to elucidate the underlying mechanisms and potential therapeutic targets involved in this process.

### Network-based analysis revealed hub genes and enriched biological processes in high glucose–treated HMrSV5 cells

**Fig. 2:**
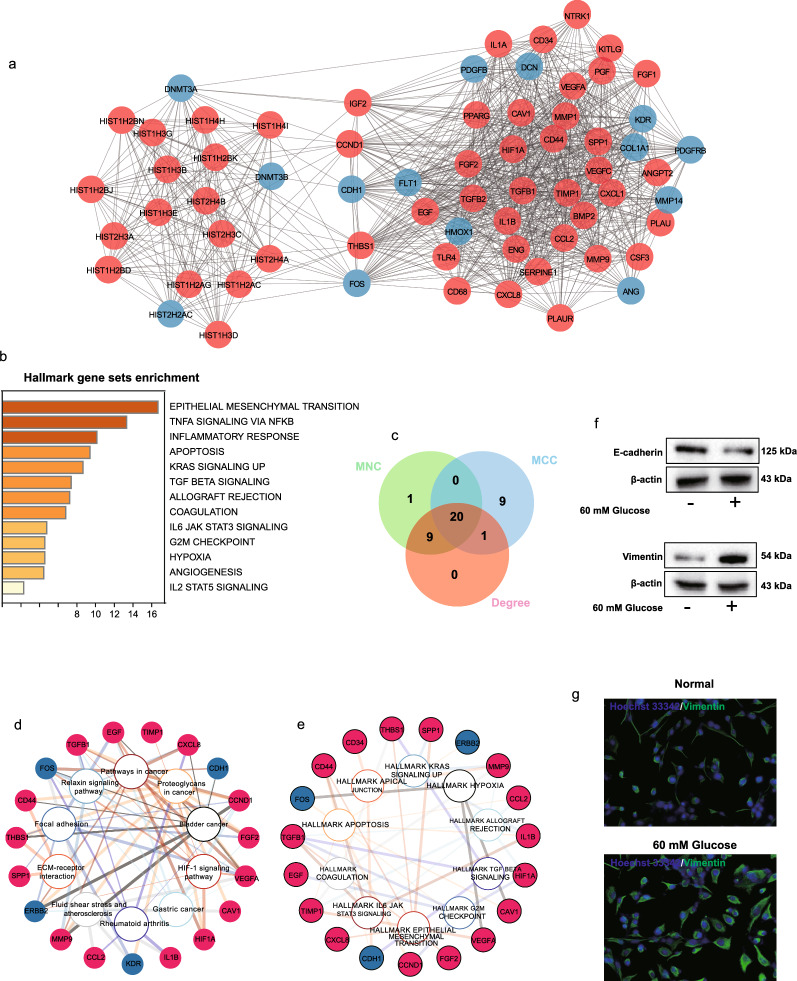
Network-based integrated analysis of DEGs. **a** The top network cluster was calculated using the MCODE app in Cytoscape. The red circle represents upregulated DEGs, while the blue circle indicates downregulated DEGs. **b** Hallmark gene set enrichment analysis of upregulated DEGs in the upper network cluster. **c** Overlap of hub genes predicted by the MNC, MCC, and Degree methods via the CytoHubba app. **d**, **e** The network shows the KEGG enrichment (**d**) and hallmark gene set enrichment (**e**), along with the genes associated with the overlapping hub genes in **c**. **f** Protein levels of E-cadherin and Vimentin in the high-glucose group compared with those in the control group. β-actin served as the protein loading control. **g** Immunostaining of Vimentin in HMrSV5 cells cultured in high-glucose medium versus control culture medium

To better understand the mechanism involved in high glucose treatment in HMrSV5 cells, we employed network-based tools, such as MCODE and CytoHubba, to explore the main subnetworks and hub genes. The analysis revealed that the highest scored subnetwork primarily consisted of upregulated DEGs, suggesting their crucial role in this process (Fig. 2a). Notably, some of the key upregulated genes in this subnetwork included *HIF-1α*, *TGF*-*β2*, *SPP1*, *FGF2*, *EGF*, *TGF*-*β1*, and *IGF2*. Additionally, an intriguing observation was the upregulation of several histone-related genes from the *HIST1H* and *HIST2H* families, suggesting the potential involvement of histone regulation in the response of HMrSV5 cells to high glucose. Furthermore, hallmark gene set enrichment analysis highlighted the functions of the upregulated DEGs in the upper subnetwork, encompassing processes such as EMT, TNF-α signaling via NF-kB, the inflammatory response, apoptosis, TGF-beta signaling, hypoxia, and angiogenesis (Fig. 2b). Considering the recognized role of EMT in fibrosis across various organs, we assessed the protein levels of E-cadherin and Vimentin through western blot and immunofluorescence staining (Fig. 2f, g). These findings indicated a decrease in E-cadherin and an increase in Vimentin, confirming the occurrence of EMT in HMrSV5 cells subjected to high glucose treatment.

Moreover, the CytoHubba results revealed 20 genes that were consistently predicted to be hub genes by multiple methods, including *FGF2*, *SPP1*, *HIF-1α*, *VEGFA*, *TGF*-*β1*, *EGF*, and *CD44* (Fig. 2c, d, e). KEGG enrichment analysis indicated that these hub genes were partially involved in ECM-receptor interactions, the HIF-1α signaling pathway, and some cancer-related pathways (Fig. 2d). Additionally, enrichment analysis of the hallmark gene sets revealed that these hub genes were enriched mainly in the pathways related to apoptosis, TGF-beta signaling, epithelial–mesenchymal transition, and hypoxia (Fig. 2e). In summary, network-based analyses emphasized the significance of upregulated genes, including key hub genes, in the response of HMrSV5 cells to high-glucose treatment. The potential involvement of histone regulation and the enrichment of diverse biological processes shed light on the intricate molecular interactions underlying high glucose-induced fibrosis and EMT in HMrSV5 cells.

### Genome-wide changes in chromatin accessibility and their links to the transcriptome

Chromosome accessible regions function as cis-DNA elements for the binding of TFs and associated proteins. The dynamic regulation of chromosome accessibility is of paramount importance in the precise control of gene expression [33]. To investigate the regulatory effect of high glucose treatment on HMrSV5 cells, we utilized ATAC-seq to profile chromosome accessibility in both control and high glucose-treated samples.

**Fig. 3:**
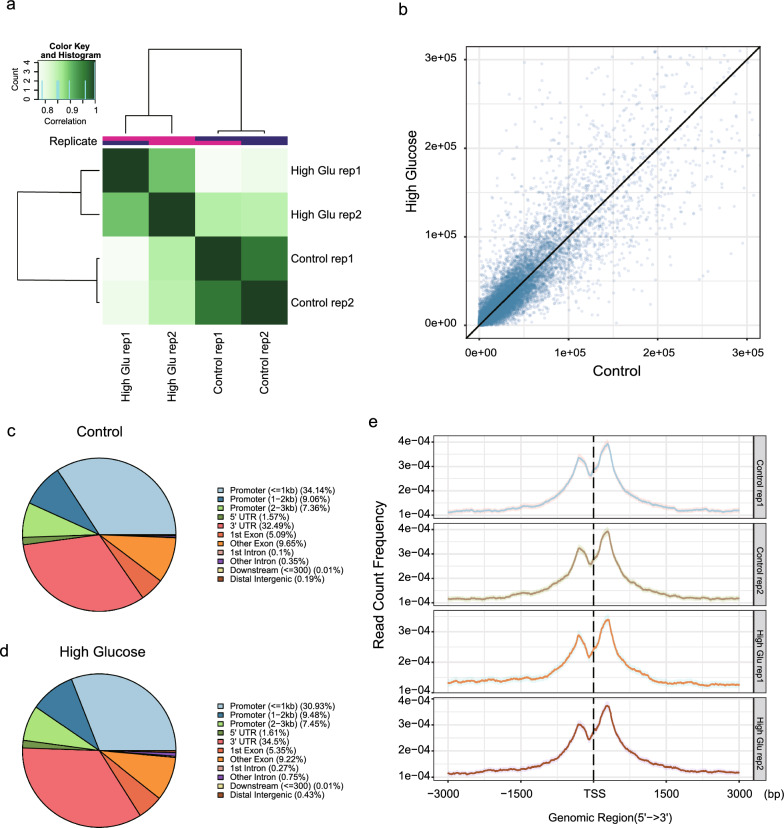
Chromosome accessibility profile of HMrSV5 under different conditions. **a** Hierarchical clustering of ATAC-seq signals from the indicated samples. **b** Correlation analysis of ATAC-seq signals between the high glucose treatment group and the control group. Each dot represents one ATAC-seq peak. The values were normalized using the following formula: R/L × 10e9, where R represents the raw read count located in the peak and L represents the total read count of the ATAC-seq library. **c**, **d** Genomic distribution of ATAC-seq peaks in control HMrSV5 cells (**c**) and high glucose–treated HMrSV5 cells (**d**). ATAC-seq signals surrounding transcript start sites (TSSs)

Hierarchical clustering of the ATAC-seq profiles revealed that the two replicates of control and high glucose-treated HMrSV5 cells were closely correlated within their respective groups, indicating good repeatability and reliability of our ATAC-seq data (Fig. 3a). Upon genome-wide analysis, we did not observe any significant overall increase or decrease in chromosome accessibility at the genomic scale (Fig. 3b). Further examination of the ATAC-seq peaks demonstrated that approximately half of these peaks were located near transcript start sites (TSSs), also known as the proximal region, in both the control and high glucose-treated samples (Fig. 3c, d). The results demonstrated that the ATAC-seq signal in the proximal region was significantly and positively correlated with the expression of the nearest genes (Additional file 2: Fig. S2a). Additionally, we observed prominent enrichment of ATAC-seq signals at regions approximately 1.5 kb surrounding the TSS, with a notably lower signal intensity directly occurring at the TSS (Fig. 3e). This observation suggested the potential binding of transcription factors at the TSS, which could be involved in the regulation of gene expression.

**Fig. 4:**
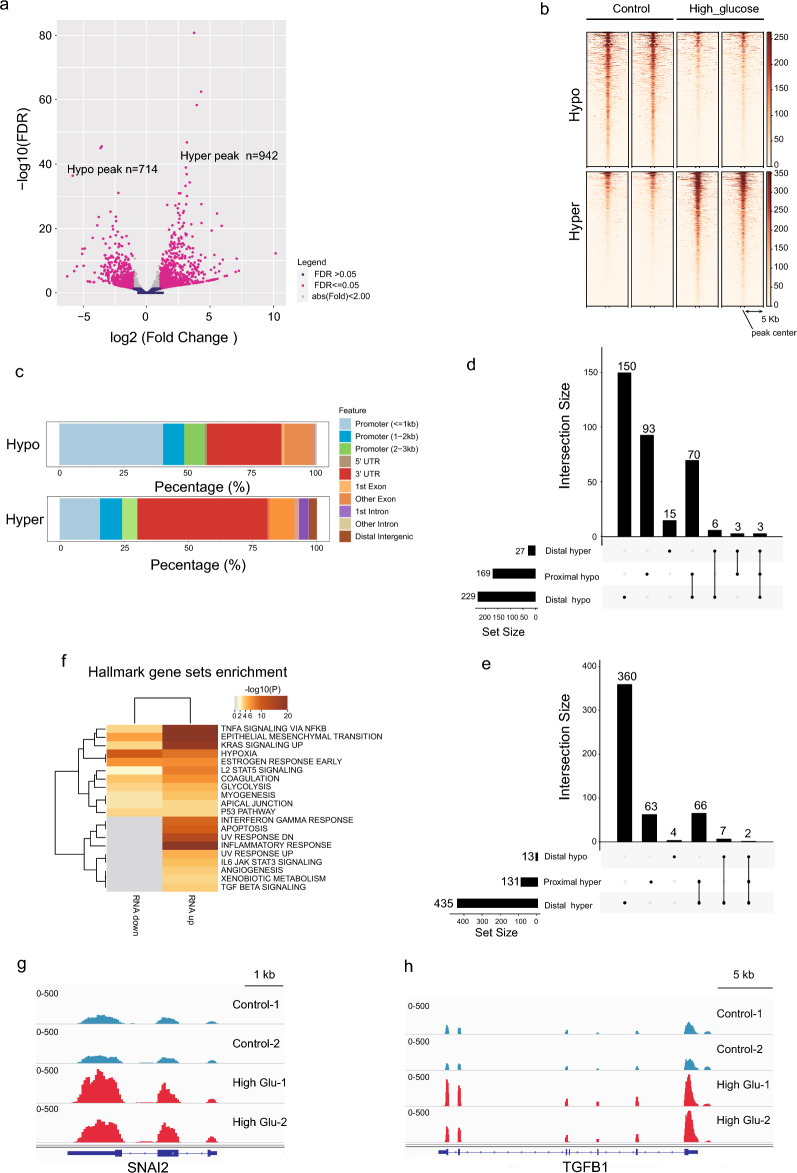
Genome-wide alterations in chromosome accessibility. **a** Volcano plots illustrating the differentially accessible regions (DAs) detected by ATAC-seq. DAs were filtered by a *P* value < 0.05 and an absolute fold change > 2. **b** Heatmap showing the ATAC-seq signals in hypo- or hyper regions. **c** Genomic distribution of hypo- and hyper-ATAC-seq peaks. **d**, **e** UpSet plot showing the association of downregulated DEGs (**d**) or upregulated DEGs (**e**) with DAs in different regions. Proximal: 2 kb region around the Transcript Start Site (TSS). Distal: region beyond 2 kb around the TSS. **f** Hallmark gene set enrichment of DAs associated with upregulated DEGs (upregulated) and downregulated DEGs (downregulated). **g**, **h** ATAC-seq signals at the loci of *SNAI2* (**g**) and *TGF*-*β1* (**h**)

To further explore the dynamics of chromosome accessibility resulting from high glucose treatment, we conducted a difference analysis to identify altered ATAC-seq peaks. Our analysis revealed 714 hypo-peaks (lower ATAC-seq signal in high glucose-treated HMrSV5 than in the control sample) and 942 hyperpeaks (higher ATAC-seq signal in high glucose-treated HMrSV5 than in the control sample) (Fig. 4a, b; Additional file 6: Supplementary table 2).

Interestingly, the hypo-peaks and hyperpeaks exhibited distinct distribution patterns. Approximately half of the hypo-peaks were located in the proximal region (within 2 kb surrounding the transcription start site), whereas only a quarter of the hyperpeaks were found in this region (Fig. 4c).

To investigate the relationship between chromosome accessibility and gene expression, we annotated the altered peaks to the closest genes when they were located in the proximal region or linked the peaks beyond the proximal region to their target genes using the GREAT tool (http://great.stanford.edu/public/html/, Additional file 7: Supplementary table 3). We observed a positive correlation between the fold change in the ATAC-seq peak and the fold change in the expression of the differentially expressed genes (DEGs) (Additional file 3: Fig. S3a, b). Additionally, hypo- or hyperpeaks located beyond the proximal region (distal region) were associated with reduced or increased expression, respectively (Additional file 3: Fig. S3b). Our comprehensive analysis revealed that approximately 398 downregulated genes were predominantly linked to hypo ATAC-seq peaks in both the proximal and distal regions (Fig. 4d). Similarly, 566 upregulated genes were annotated to hyper ATAC-seq peaks in both the proximal and distal regions (Fig. 4e). These findings suggest that alterations in chromosome accessibility play a role in the dysregulation of specific genes.

Furthermore, we focused on the upregulated genes linked to hyper ATAC-seq peaks, as they were more prominent among the main alterations observed in response to high-glucose treatment (Fig. 2). Hallmark gene set enrichment analysis demonstrated that these genes were mainly associated with TNF-α signaling via NFKB, EMT, KRAS signaling, the inflammatory response, and apoptosis (Fig. 4f). These gene sets were consistent with our previous RNA-seq analysis (Fig. 1c), further validating the hypothesis that alterations in chromosome accessibility are major epigenetic factors contributing to gene dysregulation in high glucose-induced pathological changes in HMrSV5 cells. As examples of such genes, we presented *SNAI2*, *TGF-β1*, *HIF-1α*, *FGF2*, *VEGFA*, and *VEGFC* as examples of chromosome accessibility change-associated dysregulated genes (Fig. 4g, h, Additional file 3: Fig. S4a–d).

### Identification of HIF-1α as a key transcription factor regulating epithelial–mesenchymal transition and its role in high glucose-mediated regulation of TGF-β1

**Fig. 5:**
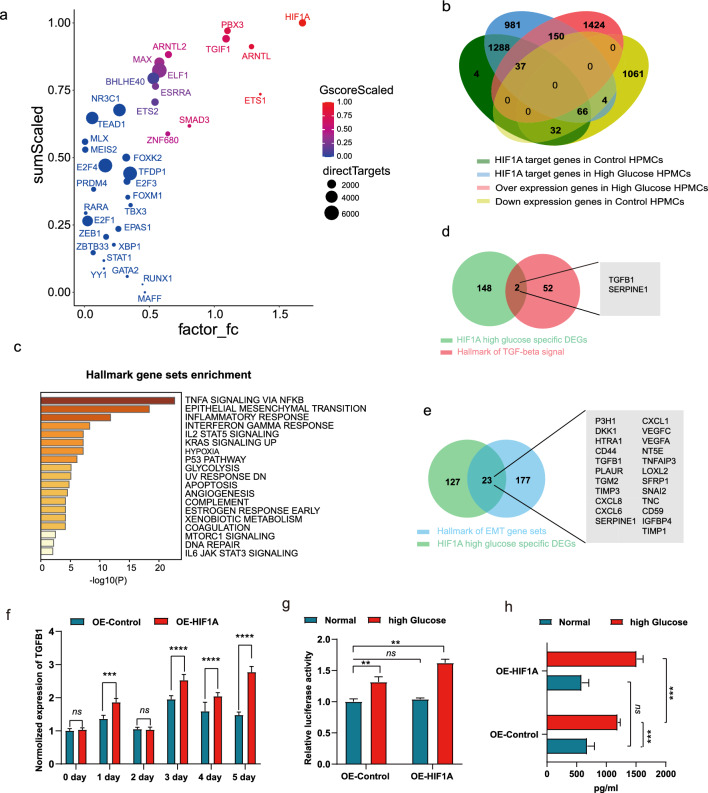
Integrated prediction and analysis of key TFs involved in high-glucose treatment. **a** Key TFs predicted through integrated analysis of ATAC-seq and RNA-seq data using ANANSE in high glucose–treated HMrSV5 cells versus controls. **b** The overlap of HIF-1α candidate target genes with differentially expressed genes in the indicated groups. **c** Hallmark gene set enrichment of HIF-1α target overexpressed genes in the high glucose-treated group. **d**, **e** HIF-1α candidate target genes in the hallmark gene sets of TGF-beta signaling (**d**) and epithelial–mesenchymal transition (EMT) (**e**). **f** Overexpression of HIF-1α exacerbates the high glucose-induced increase in *TGF-β1* mRNA expression in HMrSV5 cells. ns, not significant; *P < 0.05; **P < 0.01; ***P < 0.001. *P* values were calculated by Student’s t test. **g** Overexpression of HIF-1α exacerbates the high glucose-induced increase in the transcription of the *TGF-β1* promoter in HMrSV5 cells, as determined by dual luciferase reporter assays. **h** Quantitative assessment of TGF-β1 in the cell culture medium using ELISA

To identify potential key transcription factors associated with high glucose-treated HMrSV5 cells, we performed an integrated analysis of RNA-seq and ATAC-seq data using the ANANSE tool, which utilizes ATAC-seq data and/or H3K27ac data, along with RNA-related differentially expressed genes (DEGs), to predict key TFs. The analysis revealed 36 TFs, including HIF-1α, PRX3, TGIF1, ARNTL, and XBP1, which were predicted to play important roles in high glucose-treated HPMCs compared to those in the control treatment (Fig. 5a, Additional file 8: Supplementary table 4). Of particular interest, HIF-1α was the top candidate gene, and its association with high glucose-induced fibrosis in HMrSV5 cells has not been well documented, prompting us to further investigate its role. Next, we examined the overlap between the predicted HIF-1α target genes and the DEGs, revealing approximately 154 genes preferentially targeted by HIF-1α, the majority (150) of which were upregulated (Fig. 5b, Additional file 9: Supplementary table 5). Hallmark gene set enrichment analysis indicated that these 150 upregulated genes were enriched mainly in the TNF-α signaling pathway via NFKB, EMT, the inflammatory response, hypoxia, glycolysis, apoptosis, angiogenesis, and DNA repair (Fig. 5c). Notably, since EMT was identified as a significant pathological symptom induced by high glucose-containing peritoneal dialysis in HMrSV5 cells and since TGF-β1 was reported to be the main factor responsible for this EMT process, we further investigated the intersection of these 150 HIF-1α target genes with genes involved in the TGF-beta pathway and EMT-associated genes (Fig. 5d, e). Our analysis revealed that HIF-1α regulates *TGF-β1* and *SERPINE1* in the TGF-beta pathway, as well as 23 other genes, including *TGF*-*β1*, *CXCL8*, *CXCL6*, *SNAI2*, *VEGFA*, and *VEGFC*, which are associated with EMT.

To validate the regulatory effect of HIF-1α on TGF-β1, we established *HIF-1α*-overexpressing HMrSV5 cells and control HMrSV5 cells through lentivirus transduction. We treated these cells with culture medium containing a high concentration of glucose or ordinary culture medium for 5 days and collected the cells at regular intervals for mRNA detection. In the ordinary culture medium, there was no significant difference in *TGF*-*β1* expression between the control and *HIF-1α*-overexpressing cells. However, under high-glucose conditions, *HIF-1α*-overexpressing cells exhibited significantly greater expression of *TGF*-*β1* on day 1, day 3, day 4, and day 5 (Fig. 5f). Moreover, the dual-luciferase activity assay corroborated the enhancement of *TGF-β1* transcriptional activity by HIF-1α in a high glucose-dependent manner (Fig. 5g). Consistently, this regulatory mechanism was confirmed by ELISA (Fig. 5h).

**Fig. 6:**
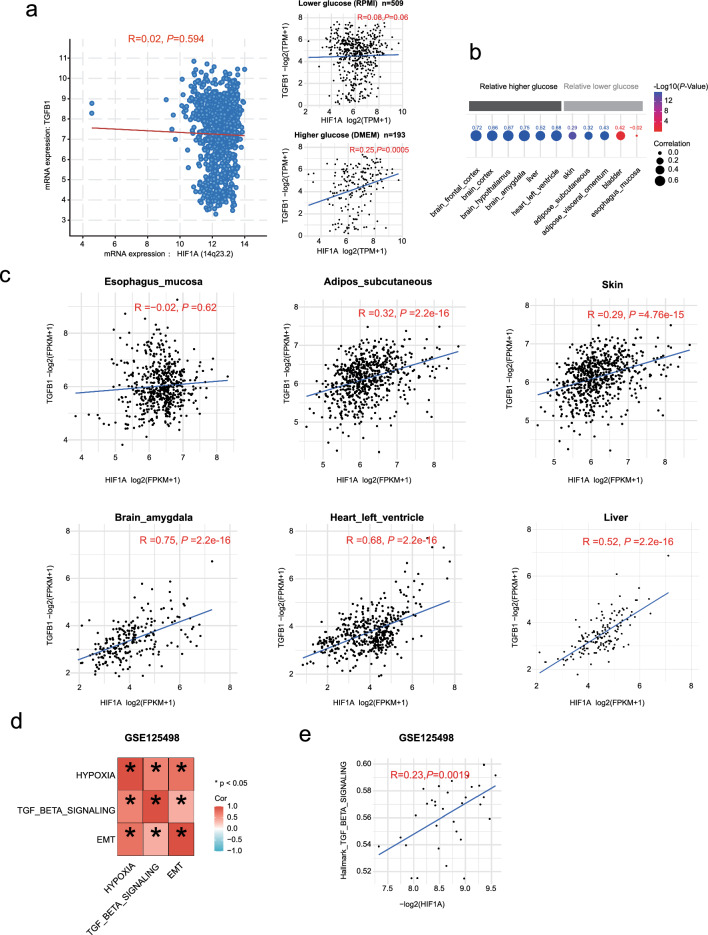
Correlations between *HIF-1α* and *TGF*-*β1* in model cells and human tissues. ***a*** mRNA expression correlation between *HIF-1α* and *TGF*-*β1* in the CCLE database. Left: all cells. Right: Subgroups of cells cultured in low-glucose culture medium or high-glucose culture medium. (https://sites.broadinstitute.org/ccle/). **b** Overview of the correlation between *HIF-1α* and *TGF*-*β1* mRNA expression in tissues with higher glucose demand or lower glucose demand. The data were sourced from the GTEx database. (https://www.gtexportal.org/). **c** Correlations of *HIF-1α* and *TGF*-*β1* expression in various tissues: the esophagus, adipose tissue, skin, brain, heart, and liver. **d** The correlation analysis between the indicated gene sets in GSE125498. **e** Positive correlation between HIF-1α and TGF-beta pathway in GSE125498

We also investigated the correlation between *HIF-1α* and *TGF*-*β1* expression using data from both the CCLE database and the GTEx database [34]. In general, *HIF-1α* and *TGF*-*β1* were not significantly associated with each other (*P* = 0.594) according to the CCLE database. However, upon closer examination, we observed a significant positive correlation between the expression of these genes in cells cultured in DMEM (R = 0.25, *P* = 0.0005, 4.5 g/L glucose) but not in cells cultured in RPMI medium (R = 0.08, *P* = 0.06, 2.05 g/L glucose) (Fig. 6a). Furthermore, we detected a stronger correlation between *HIF-1α* and *TGF*-*β1* in tissues with higher glucose demand, such as the brain, liver, and heart, than in tissues with lower glucose demand, including the skin, adipose tissue, bladder, and esophagus (Fig. 6b, c). Moreover, in the GSE125498 dataset, comprising 33 peritoneal cell samples derived from peritoneal dialysis patients, we observed a positive correlation between the hallmarks of hypoxia, the TGF-beta signaling pathway, and EMT (Fig. 6d). Additionally, we discovered a positive correlation between the expression of *HIF-1α* and the expression of genes in the TGF-beta signaling pathway (Fig. 6e). These results further support our finding that HIF-1α*-*mediated regulation of TGF-β1 is influenced by high glucose levels and suggest that HIF-1α can enhance the stimulatory effect of high glucose on TGF-β1.

### Drug discovery for peritoneal fibrosis and molecular docking of drugs with proteins

**Fig. 7:**
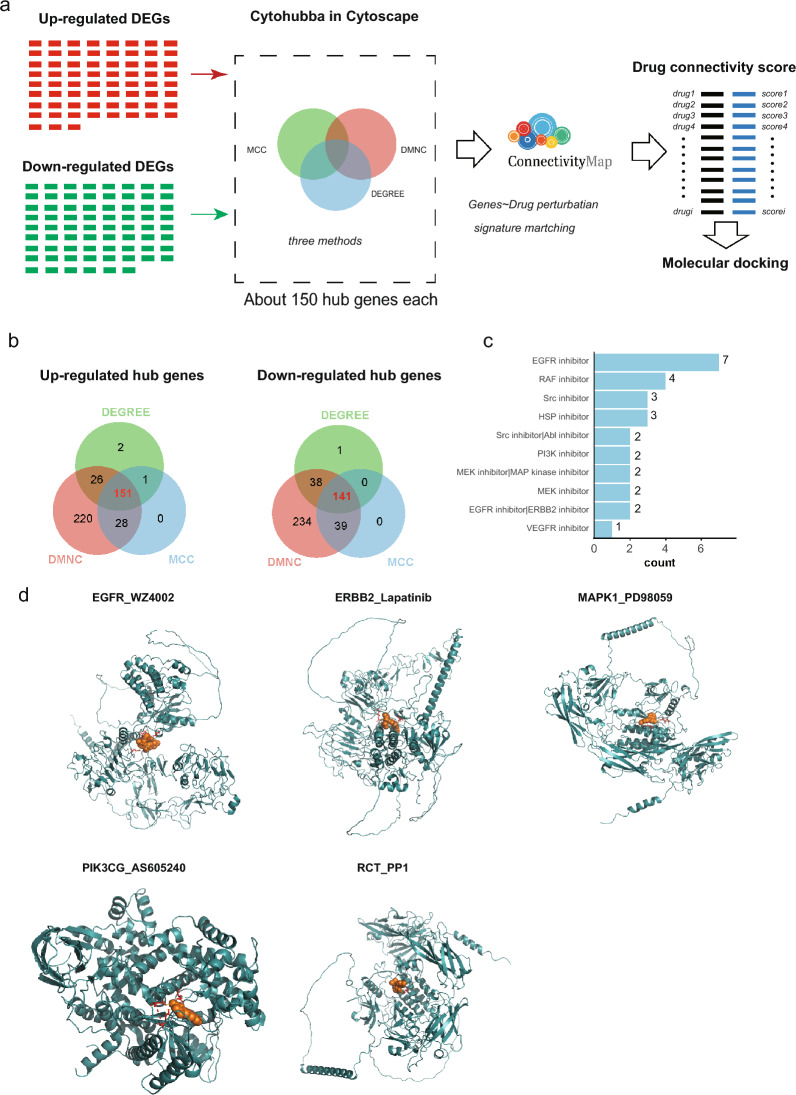
Identification and analysis of potential therapeutic agents for treating peritoneal fibrosis. **a** Flowchart for conducting drug discovery and analysis. **b** Venn diagram showing the use of the three methods for identifying hub genes for CMap analysis. **c** Number of potential drugs assigned to target pathways. **d** Molecular docking results of potential drugs to their target proteins, including WZ4002 with EGFR, lapatinib with ERBB2, PD98059 with MAPK1, AS605240 with PIK3CG, and PP1 with RCT. Orange represents the drugs, red represents the amino acids with which the drugs interact, and cyan represents the proteins

In the pursuit of identifying potential drugs for PF treatment, we conducted CMap analysis (Fig. 7a). The CMap database (connectivity map, https://clue.io) is a valuable resource for revealing the functional relationships among genes, drugs, and diseases. The dataset includes expression data from cultured human cells treated with diverse small molecules, including FDA-approved drugs and experimental compounds. CMap allows researchers to compare gene expression profiles across various experiments, aiding in the identification of drugs with similar or opposing effects on gene expression. This, in turn, facilitates the discovery of new therapeutic applications for existing drugs or the potential identification of side effects. Leveraging the three methods with Cytohubba in Cytoscape, we identified 151 commonly upregulated hub genes and 141 commonly downregulated genes, which were employed as inputs for CMap analysis on the website (Fig. 7b). We identified 73 drugs with negative connectivity scores (Additional file 10: Supplementary table 6), indicating potential efficacy in reversing PF triggered by high glucose. Notably, the top three pathways associated with these drugs were the EGFR pathway, RAF pathway, and Src pathway (Fig. 7c). Additionally, five drugs, WZ4002, lapatinib, PD98059, AS605240, and PP1, had negative scores in more than two tested cell lines. Finally, we validated the interactions of WZ4002 with EGFR, lapatinib with ERBB2, PD98059 with MAPK1, AS605240 with PIK3CG, and PP1 with RCT (Fig. 7d) with molecular docking analysis.

## Discussion

An increasing number of studies have provided evidence that high glucose levels play a pivotal role in driving peritoneal fibrosis. However, despite the involvement of several molecules in this process being previously reported [35–39], our understanding of the precise transcriptome alterations and the involvement of chromatin accessibility in the transcriptional regulatory networks triggered by high glucose concentrations is limited. Further investigations are warranted to comprehensively elucidate these mechanisms and improve our understanding of high glucose-induced peritoneal fibrosis.

In the present study, through an analysis of differential mRNA expression, we identified several upregulated genes associated with peritoneal fibrosis, including *TWIST1*, *TWIST2*, *ZEB1*, *ZEB2*, *SNAI1*, and *SNAI2*. Twist reportedly contributes to peritoneal fibrosis during PD treatment by regulating *YB-1* [40]. Additionally, ZEB2, known for preserving liver angioarchitecture and protecting against liver fibrosis [41], may also play a role in peritoneal fibrosis.

Furthermore, our hub gene analysis and network analysis identified several critical hub genes, including *HIF-1α*, *VEGFA*, *TGF-β1*, *FGF2*, *EGF*, and *MMP9*. Previous studies have suggested the importance of fibroblast growth factor (FGF) in human peritoneal mesothelial cells cultured in high glucose medium, indicating its potential significance in the initiation of peritoneal fibrosis and the possible efficacy of glucocorticoids in preventing such fibrosis in peritoneal dialysis patients [42]. Moreover, VEGF has been linked to angiogenesis, increased endothelial permeability, and angiogenesis induction [43]. EGF has been reported to induce a morphological change toward a fibroblastic phenotype [44], and EGFR has been associated with tissue fibrosis in various organs, including the liver, lung, and kidney [45–47].

Moreover, we found that these hub genes are involved in the HIF-1α signaling pathway, the IL6-JAK-STAT3 signaling pathway, hypoxia, the TGF-beta pathway, and EMT. This finding is consistent with previous studies showing that the HG/STAT3/HIF-1α signaling pathway may play an important role in the pathogenesis of peritoneal fibrosis induced by high glucose-based dialysis fluid [48]. Additionally, IL-6 has been reported to promote EMT in HPMCs possibly through the JAK/STAT3 signaling pathway [49]. Interestingly, we also observed the upregulation of multiple histone-associated genes, warranting further inquiry to elucidate the intricate role of histone regulation in the initiation of high glucose-induced peritoneal fibrosis.

The use of Tn5 transposase-based ATAC-seq offers several advantages, including increased speed, the ability to work with low input cell numbers, and improved repeatability when compared to those of FAIRE-seq and DNase-seq. By employing ATAC-seq, we reliably detected changes in chromosome accessibility during high glucose treatment in HPMCs. Our observations revealed significant alterations in the accessibility of numerous chromosome regions, with a distinct pattern characterized by hyperpeaks primarily located in distal regions. These findings suggest that long-range regulation by enhancers may play a crucial role in the upregulation of the majority of genes. For instance, the elevation of many genes, such as *TGF-β1*, *SNAI2*, and *HIF-1α*, was associated with hyper ATAC-seq peaks surrounding these genes. Previous studies have reported that Snail contributes to pancreatic tumor development by promoting fibrotic reactions through the increased TGF-beta pathway [50]. Additionally, overexpression of *TGF*-*β1* via a viral vector induces the expression of genes associated with peritoneal fibrosis and EMT [51]. Furthermore, HIF-1 is a heterodimeric protein composed of HIF-1α and HIF-1β subunits that activate the transcription of many genes encoding proteins involved in angiogenesis, extracellular matrix remodeling, migration, invasion, and metastasis [52]. As anticipated, the identified chromosome accessibility-associated genes are largely in line with the alterations observed in the transcriptome, suggesting that chromosome accessibility alterations predominantly play a role in the modification of genes induced by high glucose. Open chromatin regions function as crucial cis-regulatory elements, facilitating the binding of transcription factors. Therefore, our findings offer valuable insights for advancing the understanding of the regulatory mechanisms governing these genes.

Open chromatin regions play crucial roles as cis-regulatory elements by facilitating the binding of transcription factors. Through the integration of chromosome accessibility and RNA-seq data, we identified several key transcription factors involved in the regulation of gene changes triggered by high glucose, including *SMAD3*, *PBX3*, *ARNTL*, and *E2F4*. *ARNTL2* knockdown reportedly suppressed EMT activity, as evidenced by reduced expression of N-cadherin and Vimentin and increased expression of E-cadherin [53]. In addition, the Snail-Smad3/TGF-beta signaling pathway synergistically augments EMT and migration in hepatocellular carcinoma (HCC) [54].

Furthermore, we made an intriguing observation that HIF-1α appears to potentially regulate TGF-β1 in a high glucose-dependent manner, a finding that we subsequently validated through our experiments and dataset exploration. Although several mechanisms have been elucidated in previous research, such as PKC mediating the high glucose-induced upregulation of TGF-β1 and fibronectin synthesis by HPMC, leading to the progressive accumulation of extracellular matrix and eventual peritoneal fibrosis [55], and Ang II-induced TGF-β1 and fibronectin expression in HPMC being mediated by NADPH oxidase-dependent ROS [56], our study presents the first report of HIF-1α promoting TGF-β1 mRNA levels, promoter transcription activity and protein levels under specific conditions. These results contribute to a deeper understanding of the intricate regulatory mechanisms involved in high glucose-triggered gene changes and their implications for peritoneal fibrosis development. Several drugs have been found to have the function of inhibiting HIF-1α activity, including 2-Methoxyestradiol, Bendazol, PX-478, etc. Bendazol is an antihypertensive drug that can enhance NO synthase activity in glomeruli and collecting tubules. Bendazol can inhibit the development of form-deprivation myopia (FDM) and the upregulation of HIF-1α. PX-478 2HCl is an orally active selective HIF-1α inhibitor. PX-478 2HCl can induce apoptosis and has anti-tumor activity [57]. However, the role of these drugs in the treatment of peritoneal fibrosis remains to be further verified, and it is expected to be helpful for clinical treatment.

There are several limitations in our research that warrant acknowledgment. First, it is important to note that our study was exclusively conducted using a model cell line (HMrSV5). There are not enough samples from patients, and insufficient data have not been collected at present. To validate our findings, additional clinical samples need to be collected for further analysis. Second, additional research is necessary to fully comprehend the intricate mechanisms by which HIF-1α promotes the level of TGF-β1 under high-glucose stimulation.

## Conclusions

Our study provides a comprehensive overview of the changes in the transcriptome and chromosome accessibility in HPMCs stimulated with high glucose. Notably, we observed a strong correlation between alterations in chromosome accessibility and gene expression, highlighting the pivotal role of these alterations in the pathological changes induced by high glucose. Most importantly, we have identified crucial transcription factors participating in this process and demonstrated the regulation of *TGF-β1* by HIF-1α in a high glucose depended manner. We also explored pharmaceutical compounds that exhibit potential therapeutic efficacy against peritoneal fibrosis. Overall, our findings contribute to an enhanced understanding of the regulatory mechanisms underlying HPMC pathology changes triggered by high glucose and offer potential targets for future therapeutic interventions.

### Supplementary Information

**Additional file 1: Fig. S1.** Gene Ontology enrichment analysis and RNA expression analysis of key transcription factors associated EMT and dysregulated extracellular cytokines. **a **GO enrichment of dysregulated genes. Up/Down: upregulated or downregulated genes in high glucose–treated HMrSV5 cells. **b**, **c** RNA expression of well-known key EMT-associated TFs (**b**) or dysregulated EMT-promoting external cytokines (**c**).* ns,* not significant; *P<0.05; **P<0.01; ***P<0.001. *P* values were calculated by Student’s t test.

**Additional file 2: Fig S2.** The ATAC-seq signal in the proximal region positively correlated with the annotated gene expression. **a** The correlation between the ATAC-seq signal and the expression of the nearest genes. The correlation score was calculated by Spearman correlation analysis.

**Additional file 3: Fig S3.** Alterations in ATAC-seq signals positively correlate with gene dysregulation. **a** The correlation between the fold change in ATAC signals and the fold change in annotated DEGs (only genes with a fold change >=2 were considered). **b** Gene expression of hyper or hypo-ATAC-seq peak-annotated genes between the control and high-glucose treatment groups (p values were calculated by Student’s t test).

**Additional file 4: Fig S4.** Hyper ATAC-seq signals were detected at the *HIF-1α*, *FGF2, VEGFA*, and *VEGFC* loci. **a** ATAC-seq signals at the indicated gene loci (left) and the RNA expression of the associated gene (right).

**Additional file 5:** Supplementary table 1.

**Additional file 6:** Supplementary table 2.

**Additional file 7:** Supplementary table 3.

**Additional file 8:** Supplementary table 4.

**Additional file 9:** Supplementary table 5.

**Additional file 10:** Supplementary table 6.

## Data Availability

The study's findings are supported by the data available in the paper and its supplementary information file. Additionally, all sequencing data can be accessed through the Gene Expression Omnibus (GEO) accession number GSE2251221. For any other relevant data not available in the paper or GEO, interested parties can request access from the corresponding authors, who are subject to reasonable conditions.

## Abbreviations

HPMCs: Human peritoneal mesothelial cells
DEGs: Differentially expressed genes
EMT: Epithelial–mesenchymal transition
PD: Peritoneal dialysis
ESKD: End-stage kidney disease
PF: Peritoneal fibrosis
DARs: Differentially accessible regions
TFs: Transcription factors
Das: Accessible regions
TSS: Transcript start site
FGF: Fibroblast growth factor
KEGG: Kyoto Encyclopedia of Genes and Genomes
GO: Gene ontology
ATAC: Assay for targeting accessible-chromatin
HG: High glucose
DMEM: Dulbecco’s modified Eagle’s medium
FBS: Fetal bovine serum
TPM: Transcripts per million
MAPK: Mitogen-activated protein kinase
HIF-1α: Hypoxia inducible factor-1α
STAT3: Signal transducer and activator of transcription 3
α-SMA: α-Smooth muscle actin
TGF-β1: Transforming growth factor-β1
ACE: Angiotensin II-generating enzyme
VEGF: Vascular endothelial cell growth factor
IL-6: Interleukin-6
ELISA: Enzyme-linked immunosorbent assay
