# Supplementary figures and images for: Integrative analysis of chromatin accessibility and transcriptome landscapes in the induction of peritoneal fibrosis by high glucose
Source: J Transl Med. 2024 Mar 5;22:243. doi: 10.1186/s12967-024-05037-6 (PMC10916192; doi:10.1186/s12967-024-05037-6)

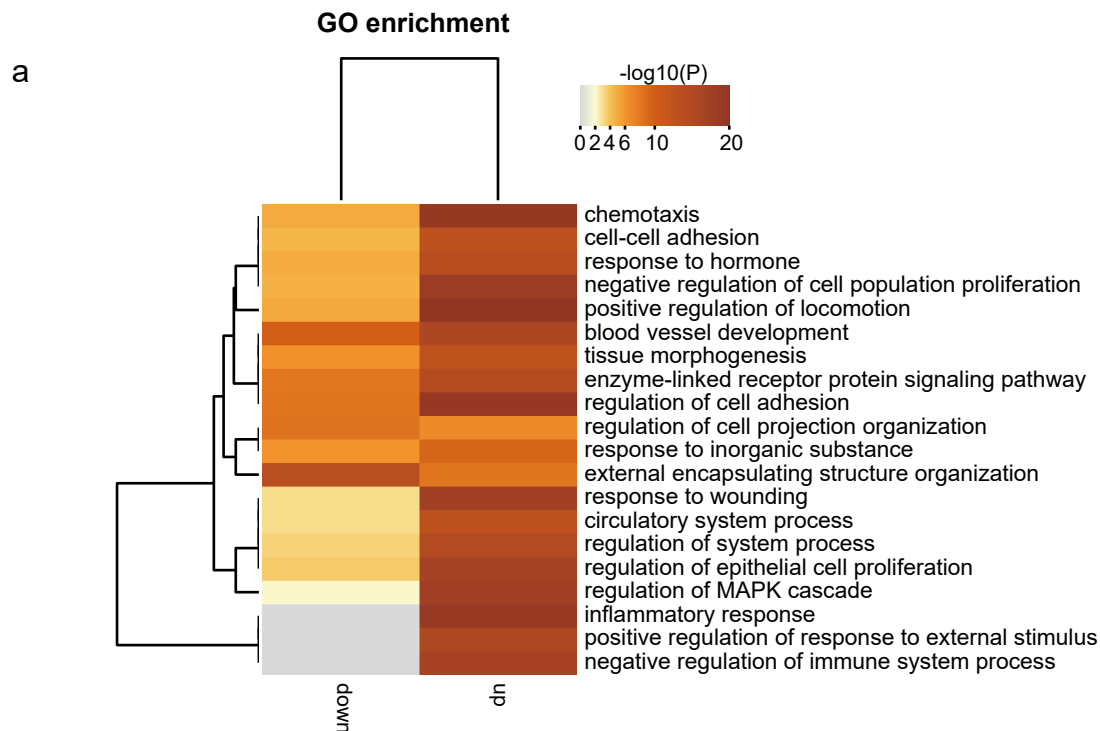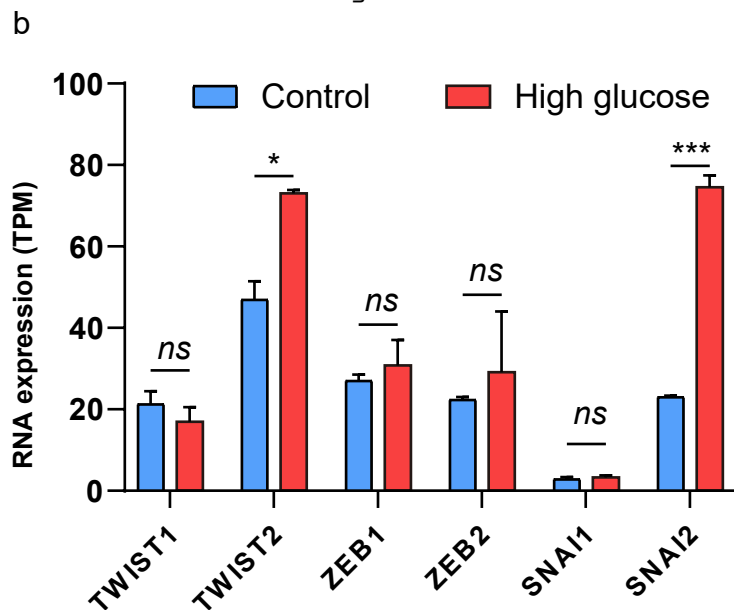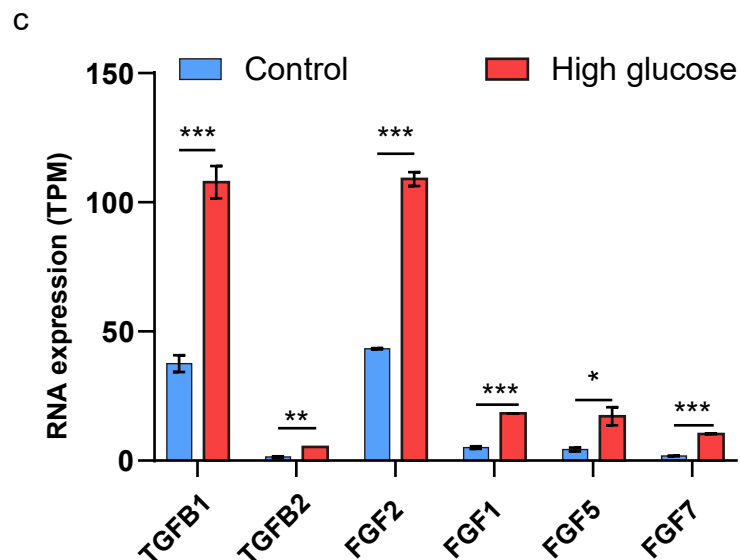

Supplement: Supplementary file 1 — Additional file 1: Fig. S1. Gene Ontology enrichment analysis and RNA expression analysis of key transcription factors associated EMT and dysregulated extracellular cytokines. a GO enrichment of dysregulated genes. Up/Down: upregulated or downregulated genes in high glucose–treated HMrSV5 cells. b, c RNA expression of well-known key EMT-associated TFs (b) or dysregulated EMT-promoting external cytokines (c). ns, not significant; *P<0.05; **P<0.01; ***P<0.001. P values were calculated by Student’s t test. [file 12967_2024_5037_MOESM1_ESM.pdf]

a

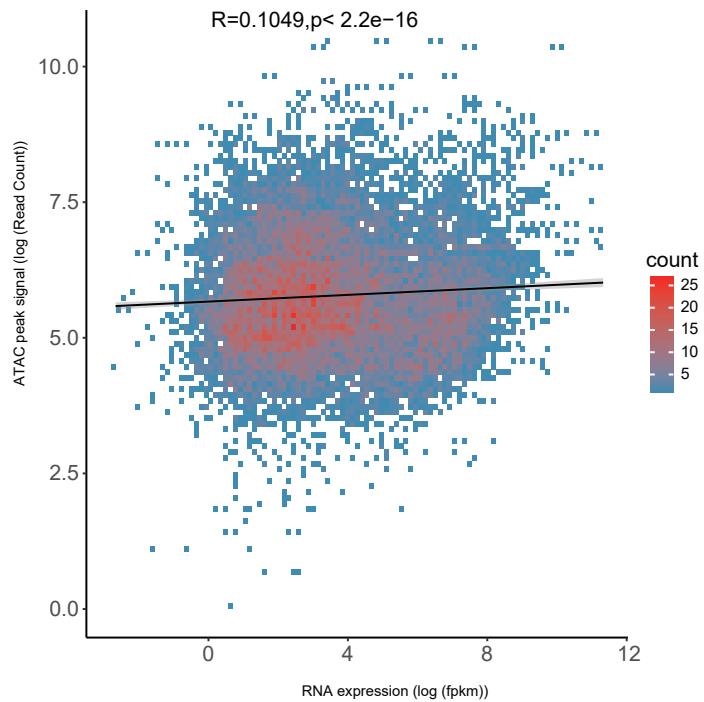

Supplement: Supplementary file 2 — Additional file 2: Fig S2. The ATAC-seq signal in the proximal region positively correlated with the annotated gene expression. a The correlation between the ATAC-seq signal and the expression of the nearest genes. The correlation score was calculated by Spearman correlation analysis. [file 12967_2024_5037_MOESM2_ESM.pdf]

a

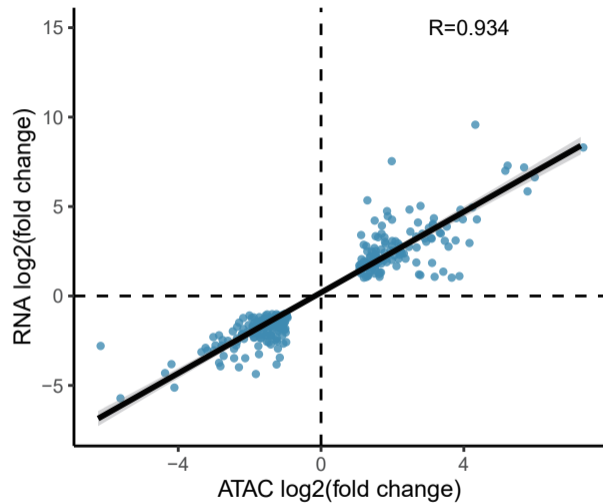

b

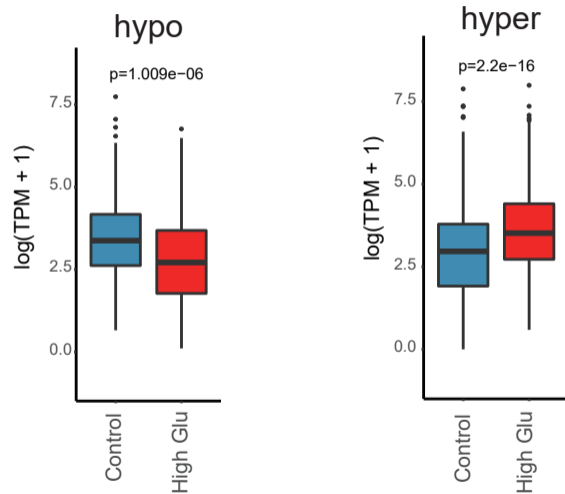

Supplement: Supplementary file 3 — Additional file 3: Fig S3. Alterations in ATAC-seq signals positively correlate with gene dysregulation. a The correlation between the fold change in ATAC signals and the fold change in annotated DEGs (only genes with a fold change >=2 were considered). b Gene expression of hyper or hypo-ATAC-seq peak-annotated genes between the control and high-glucose treatment groups (p values were calculated by Student’s t test). [file 12967_2024_5037_MOESM3_ESM.pdf]

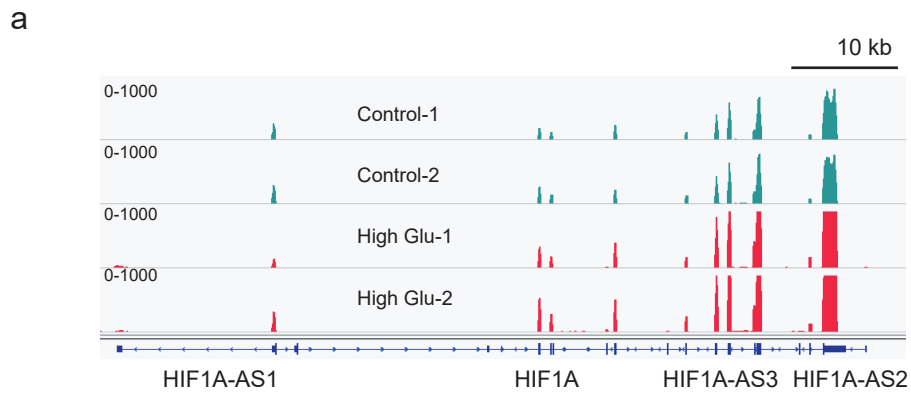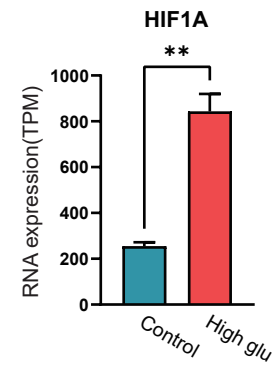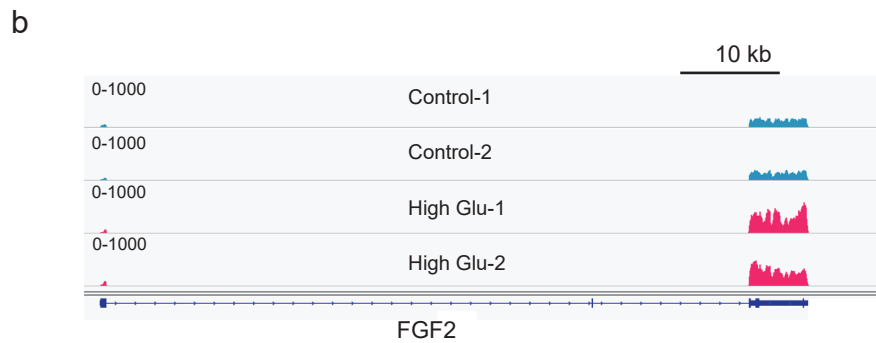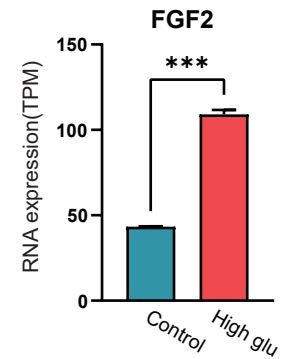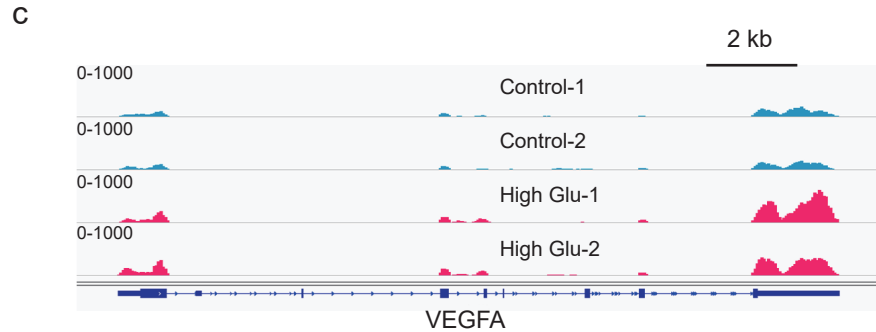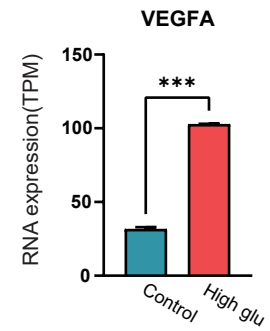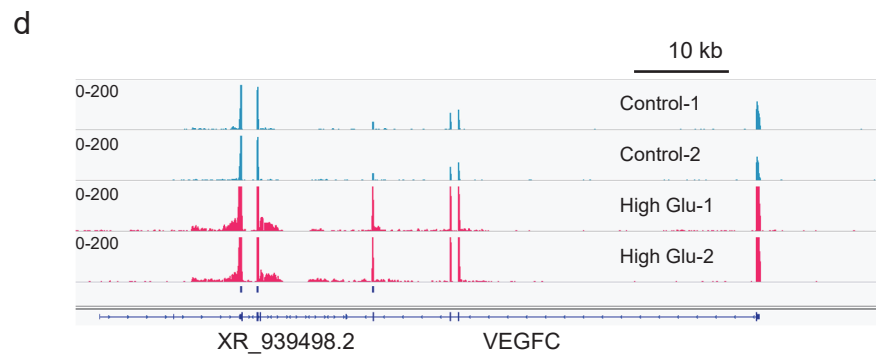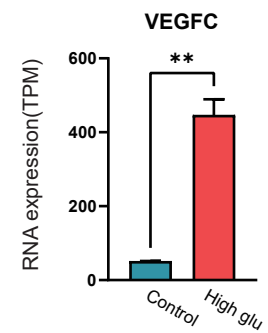

Supplement: Supplementary file 4 — Additional file 4: Fig S4. Hyper ATAC-seq signals were detected at the HIF-1α, FGF2, VEGFA, and VEGFC loci. a ATAC-seq signals at the indicated gene loci (left) and the RNA expression of the associated gene (right). [file 12967_2024_5037_MOESM4_ESM.pdf]
